# How Oxytocin Receptor (OXTR) Single Nucleotide Polymorphisms Act on Prosociality: The Mediation Role of Moral Evaluation

**DOI:** 10.3389/fpsyg.2017.00396

**Published:** 2017-03-21

**Authors:** Siyuan Shang, Nan Wu, Yanjie Su

**Affiliations:** ^1^School of Psychological and Cognitive Sciences and Beijing Key Laboratory of Behavior and Mental Health, Peking University, BeijingChina; ^2^Teachers’ College of Beijing Union University, BeijingChina

**Keywords:** moral evaluation, prosociality, oxytocin receptor gene, gender differences, mediation

## Abstract

Prosociality is related to numerous positive outcomes, and mechanisms underlying individual differences in prosociality have been widely discussed. Recently, research has found converging evidence on the influence of the oxytocin receptor (*OXTR*) gene on prosociality. Meanwhile, moral reasoning, a key precursor for social behavior, has also been associated with variability in *OXTR* gene, thus the relationship between *OXTR* and prosociality is assumed to be mediated by moral evaluation. The current study examines the relationship in question, and includes gender as a potential moderator. Self-reported prosociality on Prosocial Tendencies Measure and evaluation on the moral acceptability of behaviors in stories from 790 Chinese adolescents (32.4% boys) were analyzed for the influence of their *OXTR* single nucleotide polymorphisms (SNPs). Results showed that SNP at site rs2254298 was indirectly associated with prosocial behaviors via moral evaluation of behaviors, and this effect was moderated by gender. Our findings suggest an indirect association between genetic variations in *OXTR* and prosociality through moral evaluation, indicating the potential pathway from genetic variability to prosociality through level of moral development. We also provide some evidence that the role of oxytocin system may to some extent depend on gender. These findings may promote our understanding of the genetic and biological roots of prosociality and morality.

## Introduction

Prosociality is an umbrella term for positive emotions, attitudes, and behaviors directed toward others (Israel et al., 2015), which lead to known beneficial outcomes, including higher self-esteem, and positive social interactions (Christ et al., 2016). Attempts to better understand mechanisms underlying individual differences in prosociality have resulted in a large body of literature (Penner et al., 2005). Recently, genetic factors have been showed to contribute to such differences (Knafo et al., 2008; Christ et al., 2016), and multiple facets of prosociality had also been shown to be associated with certain common genetic determinant (Knafo-Noam et al., 2015). One factor of particular interest, the oxytocin receptor (*OXTR*) gene at the region of chromosome 3p25, which encodes the oxytocin receptor, may partly explain individual differences in social behaviors (Christ et al., 2016) and associated traits such as trust (Krueger et al., 2012). Studies show that a single nucleotide polymorphism (SNP) of this gene at rs2254298, a site in the third intron in the sequence, has impact on neuroanatomical correlates of social cognition in humans and subsequently on behavior (Brüne, 2012). For instance, Israel et al. (2009) found that in males the genetic variability at rs2254298 was associated with their performance in the Dictator Game, although the exact pattern was not mentioned. Parker et al. (2014) found that carriers of A allele at rs2254298 (AA, AG) scored worse on social functioning than those with two G alleles. Consistent with these findings, carriers of A allele at rs2254298 among Chinese (Wu et al., 2005) and Japanese individuals (Liu et al., 2010), and of G allele among Caucasian individuals (Jacob et al., 2007) were found to have a higher risk for autism, of which social impairment was a core symptom (Quintana et al., 2015). These findings indicated the influence of *OXTR* gene on many facets associated with prosociality. Research addressing underlying mechanisms has found that the influence on behavioral outcomes is possibly mediated by social cognition. Christ et al. (2016) found that variabilities in *OXTR* SNPs, including rs2254298 and rs2268498, the latter being a site in the promoter region, were indirectly associated with prosocial behaviors via empathic tendency. However, empathic tendency might not be the only intermediate and more work is needed to show how the *OXTR* gene affects prosocial behavior.

As mentioned above, to study sociocognitive factors that have more immediate influence on behavior might help understand how gene-level factors work. In particular, moral reasoning had been identified as a key precursor for prosocial behavior in both theoretical and empirical studies (Eisenberg et al., 2005; Hawley and Geldhof, 2012). The relationship between moral judgment and morality-related social behavior had been identified by meta-analysis (Wu and Liu, 2014), and fundamental studies of brain-behavior relationship also clarifies the mechanisms that link moral evaluation to prosocial behavior (Cowell and Decety, 2015). Longitudinal study also showed that moral reasoning predicted prosocial behaviors (Carlo et al., 2010). At the genetic end, the heritability of moral development has also been demonstrated by twin studies (Campbell et al., 2009). Moreover, studies have begun to show that *OXTR* polymorphisms predict individual difference in moral reasoning (Walter et al., 2012; Ohtsubo et al., 2014). Walter et al. (2012) investigated the relationship between *OXTR* gene and moral evaluation of social behavior, and found a significant difference between carriers and non-carriers of C allele at rs2268498 in the extent to which they exculpated agents for accidental harms, indicating that carriers of the C allele rated accidentally committed harm as significantly more blameworthy than non-carriers. These findings, together with evidence noticed above, suggested moral cognition and prosocial behavior probably share the same genetic and biological basis, and indicated moral evaluation of social behavior might mediate the association between *OXTR* gene and prosocial behavior. However, evidence has yet to be provided for this mediation.

In addition, the basal oxytocin level might differ in males and females (Ozsoy et al., 2009), whilst *OXTR* gene transcription and oxytocin receptor binding are regulated by sex hormones in animal models (Gimpl and Fahrenholz, 2001). Accordingly, there is substantial evidence suggesting that at least some of oxytocin receptor gene’s influences are gender-specific. For instance, some previous studies have found associations between certain *OXTR* SNPs and prosocial behaviors (Israel et al., 2009), prosocial tendency (Christ et al., 2016), or autism-associated traits (Chen and Johnson, 2012) exclusively for males, whereas some other SNPs have been linked to prosociality in females (Israel et al., 2009). Similarly, after exogenous oxytocin administration, males tended to more strongly endorse self-benefit outcomes in moral dilemmas, but females were influenced in the opposite way (Scheele et al., 2014). Thus, it is reasonable to hypothesize that gender might moderate the potential pathway from *OXTR* gene to moral evaluation and prosocial behavior.

Thus, the present study was designed to extend previous research and bridge the gap between the variability in *OXTR* gene and prosocial behavior, by examining the potential mediating role of moral evaluation on the association between *OXTR* gene and prosocial behavior. SNP rs2254298 was of primary interest as a genetic determinant to human social cognition and behavior (Brüne, 2012), and rs2268498 was to date the only *OXTR* SNP known to be linked with moral development (Walter et al., 2012). Thus, these two candidate SNPs were selected to exam this potential pathway. Finally, we included gender as a potential moderator for well-established gender-specific influence of *OXTR* gene on moral evaluation and social behavior.

## Materials and Methods

### Participants

A total of 790 Chinese adolescents (256 males, 534 females) took part in our study. All participants (*M_age_* = 16.54 years, *SD_age_* = 0.71 years) were students in a public high school in Sichuan province, non-clinical and genetically unrelated. For some unidentified reason, in most classes of the school, the majority of students were female, which is reflected in the gender composition of the resulting sample. However, the male sub-sample itself is large enough for further analysis and can be fairly representative. The present experiment was approved by the Institutional Review Board of the Department of Psychology at Peking University, and written consent was obtained from each participant and, because they were minors, from their school prior to the experiment. Participants first completed a moral story task and 3 months later they completed a paper-and-pencil version of the Prosocial Tendencies Measure (PTM) and had their buccal cells collected for genotyping. Each participant received a small gift upon completion.

### Moral Evaluation

Moral evaluation, an index of moral development, was measured with a moral story task, an applications of theory of prosocial reasoning (Eisenberg et al., 2001). A participant was presented with four pairs of realistic stories. In each pair, the protagonist just encountered a situation where he or she was in a conflict of interest with someone else and had to make a choice regarding morality. One story would end up with the protagonist choosing to commit a moral transgression, namely “Harming others,” “Stealing,” “Refusing to Help,” or “Refusing to Share,” and the other would end up with an alternative, moral choice. The stealing-or-not scenarios were adapted from previous studies (Keller et al., 2003). Participants were asked to evaluate whether the protagonist should do so on a seven-point Likert scale ranging from 0 (extremely wrong) to 6 (perfectly OK), and the four stories in which the protagonist did immorally were reverse scored. The internal reliability for this measure was good, with Cronbach’s alpha of 0.72.

### Prosocial Tendency Measure

The PTM (Carlo et al., 2003) is a 25-item version composed of the following six subscales: public (four items), anonymous (five items), dire (three items), emotional (four items), compliant (five items), and altruistic (four items). The participants were requested to rate, on a five-point scale ranging from 1 (does not describe me at all) to 5 (describes me greatly), the extent to which the statements described themselves. Previous research has demonstrated that the Chinese version of the PTM has adequate internal reliability and validity (Kou et al., 2007). In this study, the total score of PTM was used to assess prosocial behavioral tendencies in varying situations, and the internal reliability for this measure was good, with Cronbach’s alpha of 0.90.

### Genotyping

DNA was extracted from buccal cells collected by FlexiGene DNA Kit (Qiagen, Valencia, CA, USA). Two SNPs in *OXTR* gene (rs2268498 and rs2254298) were chosen in the Hapmap database. Both SNPs were genotyped using an improved multiplex ligation detection reaction (LDR) technique developed by Genesky Biotechnologies, Inc. (Shanghai, China), in which a multiplex PCR-ligase detection reaction method was used. Different SNPs were distinguished by different extended lengths at the 3′ end, and the alleles in each SNP were distinguished by different fluorescent labels of allele-specific oligonucleotide probe pairs. All primers, probes and labeling oligos were designed by and ordered from Genesky Biotechnologies, Inc. (Shanghai, China). The raw data was analyzed by GeneMapper 4.1. A random DNA sample from 5% of participants was genotyped twice for quality control, and yielded a reproducibility of 100%. **Table 1** summarizes the minor allele frequencies (mAFs) and *p*-values for the Hardy–Weinberg equilibrium tests. All allele frequencies were in Hardy–Weinberg Equilibrium and showed low linkage disequilibrium (*r^2^* = 0.094; *D′* = 0.72).

**Table 1:** Oxytocin receptor (*OXTR*) SNP genotype frequency.

SNP	Genotype	mAF	Frequency	*n*	Total	*p*-HWE
rs2254298	AA/GA/GG	A = 0.291	0.08/0.42/0.50	65/330/395	790	0.735
rs2268498	CC/CT/TT	C = 0.301	0.08/0.44/0.48	66/344/380	790	0.335

### Statistical Analysis

The sample was tested for Hardy–Weinberg equilibrium with MS-Excel. The following work was done with SPSS 22.0 for windows. Descriptive statistics for prosocial tendency and moral evaluation by genotype and gender were calculated. Analyses of variance (ANOVAs) were run to show the effects of gender, the genotype of *OXTR* SNPs (three types for each site), and their interaction on prosocial tendency and moral evaluation. Finally, the mediating role of moral evaluation was tested for with the PROCESS plug-in. Since there are three different genotypes on each SNP, we recoded each SNP into two dummy variables (at rs2268498, 1/0 for the presence/absence of C or T each; at rs2254298, the same codes with A or G) and tested their association with prosocial tendency through moral evaluation, using linear regression analysis. And for gender, males were coded as 1 and females were coded as 0. Sobel’s test and bootstrapping method had been used to examine the significance of mediation effect. As noted above, two types of behavior – moral and immoral – were evaluated, but the two subsets of data yielded a similar pattern of results with respect to the role of moral evaluation. Thus, a combined score of moral evaluation for each participant was used. Separate analyses of evaluation of moral and immoral behavior are presented in the Supplementary Material.

## Results

The average evaluation of morality issues was 4.79 (*SD* = 0.77; range = 0.00–6.00), with higher scores representing a more prosocial pattern of judgment, by which moral behavior was judged more affirmatively and immoral behavior more harshly. The average score of prosocial tendency was 3.57 (*SD* = 0.47; range = 1.00–5.00), with higher scores representing a stronger tendency (see **Table 2**). Moral evaluation of behaviors is positively related to prosocial tendency (*r* = 0.341, *p* < 0.001).

**Table 2:** Prosocial tendency and moral evaluation by genotype and gender.

			Females		Males		Total	
			*n*	*M (SD)*	*n*	*M (SD)*	*N*	*M (SD)*
Prosocial tendency	rs2254298	AA	43	3.67 (0.46)	22	3.29 (0.61)	65	3.54 (0.54)
		GA	211	3.54 (0.42)	119	3.64 (0.45)	330	3.58 (0.44)
		GG	280	3.55 (0.47)	115	3.59 (0.51)	395	3.56 (0.48)
	
	rs2268498	CC	47	3.58 (0.45)	19	3.67 (0.62)	66	3.60 (0.50)
		CT	232	3.56 (0.46)	112	3.57 (0.49)	344	3.56 (0.47)
		TT	255	3.55 (0.44)	125	3.59 (0.50)	380	3.57 (0.46)
	
Moral evaluation	rs2254298	AA	43	5.23 (0.59)	22	4.08 (0.96)	65	4.77 (0.89)
		GA	211	4.88 (0.67)	119	4.67 (0.79)	330	4.81 (0.72)
		GG	280	4.87 (0.69)	115	4.54 (0.97)	395	4.77 (0.80)
	
	rs2268498	CC	47	4.84 (0.71)	19	4.17 (1.04)	66	4.64 (0.86)
		CT	232	4.93 (0.67)	112	4.64 (0.81)	344	4.83 (0.73)
		TT	255	4.87 (0.68)	125	4.55 (0.95)	380	4.77 (0.79)

First, the effects of two SNPs in the *OXTR* gene, gender, and their interactions on prosocial tendency and moral evaluation were analyzed with ANOVAs and are shown in **Table 3**. For rs2268498, there was a significant main effect of genotype on moral evaluation, *F*(2,784) = 3.27, *p* = 0.038, $\eta_{P}^{2}$ = 0.008; follow-up pairwise comparisons showed that individuals with CT genotype judged morality issues significantly more prosocially than those with CC genotype (Cohen’s *d* = 0.238). For rs2254298, there was a significant interaction between gender and genotype on prosocial tendency, *F*(2,784) = 6.67, *p* = 0.001, $\eta_{P}^{2}$ = 0.017, and another on moral evaluation, *F*(2,784) = 7.50, *p* = 0.001, $\eta_{P}^{2}$ = 0.019. Simple effect tests showed that only in males did carriers of these three genotypes differ in prosocial tendency [*Bonferroni test*, *F*(2,784) = 5.42, *p* = 0.005, $\eta_{P}^{2}$ = 0.014] and moral evaluation [*Bonferroni test*, *F*(2,784) = 5.81, *p* = 0.003, $\eta_{P}^{2}$ = 0.015]. Specifically, male G allele carriers (GA and GG) showed more prosocial tendency (GA vs. AA: *p* = 0.003, Cohen’s *d* = 0.653; GG vs. AA: *p* = 0.015, Cohen’s *d* = 0.534) and judged morality issues more prosocially as well (GA vs. AA: *p* = 0.002, Cohen’s *d* = 0.671; GG vs. AA: *p* = 0.026, Cohen’s *d* = 0.477). There was no interaction between the two SNPs on either dependent variable.

**Table 3:** Analysis of variance (ANOVA) results for the interaction of genotype and gender on prosocial tendency and moral evaluation.

		Genotype × Gender			Genotype			Gender		
		*F*	*p*	$\eta_{P}^{2}$	*F*	*p*	$\eta_{P}^{2}$	*F*	*p*	$\eta_{P}^{2}$
Prosocial tendency	rs2254298	6.67	0.001	0.017	1.43	0.239	0.004	3.03	0.082	0.004
	rs2268498	0.19	0.830	<0.001	0.36	0.697	0.001	0.92	0.339	0.001
Moral evaluation	rs2254298	7.50	0.001	0.019	1.64	0.195	0.004	47.21	<0.001	0.057
	rs2268498	1.44	0.238	0.004	3.27	0.038	0.008	28.76	<0.001	0.035

Then, multiple linear regression was used to investigate whether moral evaluation mediated the significant influence of gender, *OXTR* SNP (rs2254298) and their interaction on prosocial behaviors. The results were in line with the ANOVA results (see **Table 4**). Significant interactions between gender and the presence of G allele were found on both prosocial tendency and moral evaluation, and females gave higher moral evaluation. In line with ANOVA results, the interactions between gender and genotype on the two measures showed that only in males could genotype be a significant predictor for each measure. In the model on prosociality concerning the hypothesized mediator role of moral evaluation, interaction between rs2254298 genotype and gender on prosocial tendency was reduced when moral evaluation was included, *Sobel’s Z* = 3.58, *p* < 0.001. This indicated a mediating effect via moral evaluation on prosocial tendency, which had also been proved by bootstrapping method (Preacher and Hayes, 2008), 95% *CI* of coefficient = [0.01, 0.04]. Plus, this was a partial mediation, as after the effect of moral evaluation was partialled out, the interaction between genotype and gender was still significant.

**Table 4:** Regression analysis with gender, rs2254298A, rs2254298G, and moral evaluation predicting prosocial tendency.

	Moral evaluation			Prosocial tendency		
	β	*t*	*ΔR^2^*	β	*t*	*ΔR^2^*
Step 1			0.041***			0.001
Gender	-0.20	-5.78***		0.03	0.84	
Step 2			0.001			<0.001
rs2254298A	0.04	1.04		0.01	0.32	
rs2254298G	0.02	0.42		0.02	0.56	
Step 3			0.018**			0.017**
rs2254298A × gender	0.03	0.95		0.03	0.83	
rs2254298G × gender	0.14	3.87***		0.14	3.65***	
Step 4			-			0.114***
Moral evaluation	-	-		0.35	10.16***	

**p < 0.01, ***p < 0.001.

**FIGURE 1:**
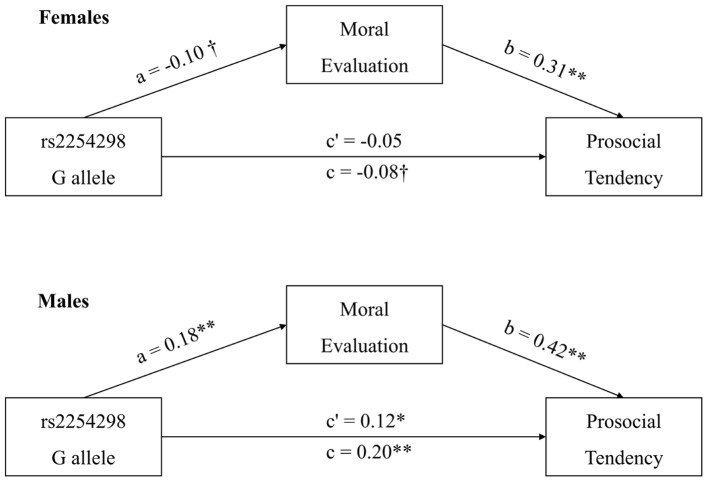
**Gender-specific effects of *OXTR* rs2254298 genotype on prosocial tendency mediated by moral evaluation.** The mediation effects of moral evaluation in both genders were tested separately. Results showed that moral evaluation mediated the relationship between the presence of G allele and prosocial tendency in both genders, but *OXTR* rs2254298 G allele influenced differently in males and females. ^†^*p* < 0.10, *p < 0.05, **p < 0.01.

Finally, to test whether and how gender moderated the mediation from rs2254298 genotype via moral evaluation on prosocial tendency, the mediating effect was tested by gender. The coefficients for the indirect effect via moral evaluation in both genders were significantly different from zero (95% *CI* = [0.04, 0.26] for males and [-0.10, -0.01] for females). However, the direction of effect varied with gender, as the plus/minus signs above implied. For males, carriers of G allele at rs2254298 exhibited a more prosocial attitude in moral evaluation task and a stronger prosocial tendency than non-carriers, whereas for females, opposite patterns were observed. These mediation patterns are illustrated in **Figure 1**.

## Discussion

The present study tested the association between the variability in *OXTR* SNPs and both prosocial tendency and moral evaluation, and explored the mediating role of moral evaluation. The results showed that two *OXTR* gene SNPs tested (rs2268498 and rs2254298) were directly related to moral evaluation and the variability in *OXTR* SNP rs2254298 was indirectly associated with prosocial behaviors via moral evaluation of behaviors, and the latter effect was moderated by gender. These findings, coupled with previous evidence showing the relationship between *OXTR* gene and both prosociality (Christ et al., 2016) and moral evaluation (Walter et al., 2012), indicated prosociality and moral cognition might share similar genetic basis. Moreover, our findings suggested that moral evaluation played a mediating role in the association between *OXTR* gene and prosociality, indicating a genetic pathway to prosociality through moral cognition. However, as ANOVA results show, the effect sizes are relatively small, and the findings should be interpreted more cautiously.

In the present study, we found the association between *OXTR* rs2254298 and both moral evaluation and prosocial tendency. The differences between three genotype groups were found to be present only in male participants. The carriers of both GG and GA genotypes were found to be more prosocial and judge morality issues more prosocially than AA genotype carriers. In line with previous studies, G allele carriers were also noted to be better at perspective taking skills (Wu et al., 2012). However, we did not fully replicate the findings of Walter et al. (2012), who reported carriers of the C allele of *OXTR* rs2268498 judged morality issues more prosocially than non-carriers, whereas we found the carriers of the CC genotype of rs2268498 judged morality issues significantly less prosocially than the T allele carriers. An explanation for this incongruence lies in the demographic differences. Specifically, participants in the Walter et al. (2012) study were all Caucasian adults, whereas the participants in our study were Chinese adolescents. Previous studies have reported the difference in allele frequency of *OXTR* SNPs between Caucasian and Asian populations (Thompson et al., 2011). Moreover, similar population-specific findings have also been found on other SNPs. For example, rs2254298 A allele has been associated with autism spectrum disorders in Chinese (Wu et al., 2005) and Japanese populations (Liu et al., 2010), while G allele is reported to be the risk allele in Caucasian populations (Jacob et al., 2007). Thus, the inconsistent findings between the two studies may be due to differential distribution and function of *OXTR* SNPs in the two races. It is also noteworthy that the sample in the current study was in adolescence, in which social cognitive skills undergo rapid development and the evaluation of social behaviors is of great importance in social interaction (Eisenberg et al., 2001; Steinberg and Morris, 2001). It is not clear how these developmental changes could interplay with genetic factors. Thus, more data would be needed to figure out whether the inconsistence resulted from racial or age differences, or both.

The present study also revealed effects in opposite directions of the *OXTR* gene (SNP rs2254298) in male and female participants. Only in male participants, G allele carriers judged morality issues significantly more prosocially and in turn tended to be more prosocial than carriers of AA genotype. By contrast, in female participants, the influence of rs2254298 was marginally significant, with G allele carriers judged morality issues less prosocially and tended to be less prosocial than carriers of AA genotype. These results add to previous findings showing gender-specific influences of *OXTR* gene and exogenous oxytocin administration (Scheele et al., 2014; Yao et al., 2014). Specifically, Christ et al. (2016) observed an association between rs2254298 and prosocial tendency only in male participants that GG genotype was associated with more prosocial behavior. Chen and Johnson (2012) reported that A allele on rs2254298 was associated with autism-associated traits in male participants, but not in female participants. Similarly, exogenous oxytocin administration also had opposing effects on moral evaluation in males and females (Scheele et al., 2014). However, the mechanisms underlying the gender-dimorphic findings remain under debate. Some research hypothesized that such dimorphism might be associated with differences in basal oxytocin level (Ozsoy et al., 2009) and oxytocin receptor distributions (Scheele et al., 2014) in males and females, yet some other studies found no gender difference in plasma oxytocin level (Hoge et al., 2008; Feldman et al., 2012). Sex hormones might also influence the transcription of *OXTR* gene and regulate oxytocin receptor binding, but again, the mechanisms of regulation remain to be understood (Gimpl and Fahrenholz, 2001). Nevertheless, the current study adds some evidence for the gender-specific influence of oxytocin system, and future studies are needed to explore the underlying mechanisms.

Although the assessment of moral evaluation in the present study could not rule out the influence of implicit emotional response (Greene, 2009), and the partial mediation of moral evaluation implies the existence of other mediator(s), our finding, together with some previous evidence (Guastella et al., 2008; Andari et al., 2010; Scheele et al., 2014), suggested that oxytocin system may have its influence on prosociality partly via a cognitive mechanism. Specifically, Scheele et al. (2014) found that exogenous oxytocin administration facilitated neural responses in ACC, precuneus and insula without corresponding impact on emotional arousal, which suggested the activation in these areas might not reflect an enhanced emotional response, but increased attention to certain type of social cues. In line with this, exogenous oxytocin administration (Guastella et al., 2008; Andari et al., 2010) and the variability in *OXTR* gene (Melchers et al., 2015) have been related to social perception or attention, which can also influence moral judgment (Pärnamets et al., 2015). Additionally, at the brain activity level, *OXTR* gene polymorphisms have been found to be associated with the functionality of amygdala (Waller et al., 2016), which plays a pivotal role in facilitating attention to and perception of salient stimuli (Phelps and LeDoux, 2005). At the molecular level, recent research (Reuter et al., 2016) has shown that C allele at rs2268498 is associated with a higher level of mRNA expression. Taken together, the literature lends support to a pathway from *OXTR* gene to brain function, perception, social affect, cognition, and finally to behavior. However, how the pathway operates is far from being fully understood, especially at the biological end, and further studies are needed to explore the complex mechanism underlying prosociality and morality (Israel et al., 2015).

Some limitations should be noted. First, the functionality of *OXTR* rs2254298 is not fully understood, especially its interaction with some other genetic variability and the environment. Thus, our study only focused on the single-gene paradigms, and future research should test multi-gene interactive effect and environmental influence as well. Second, although we had a sample large enough for a genetics study, allowing for testing the differences between three genotype groups, there are large differences in sample size between subgroups, with only 22 male participants carrying AA genotype at rs2254298. Thus, we randomly chose 22 participants from the each subgroup to conduct another ANOVA and analysis of mediation. The nature of results remained the same, as the interactions between gender and the genotype of rs2254298 on prosocial tendency and moral evaluation were still significant, and the mediation effect had also been shown with bootstrapping method. But the sample size of this randomly chosen sample is relatively small for an analysis of mediation, and becomes all the more undersized for the moderation by gender. Thus, it is essential to replicate our observations in independent samples and to extend such findings in other ethnic and age groups, which may help improve the understanding of psychosocial pathways through which individual differences in prosociality may emerge. Finally, the study was not designed to test the possibility of reciprocal influence between moral development and prosocial behaviors, which then could be a mediator on the former. More work should be done to reveal a more detailed relationship between the two measures of prosociality and morality, and their association with *OXTR* gene.

## Conclusion

The present study identifies *OXTR* SNPs as genetic contributors to both prosociality and moral cognition, and provides evidence for the pathway from *OXTR* gene to moral evaluation to prosociality, as well as some more evidence for the gender-specific influence of *OXTR* gene. Although it is still unclear how *OXTR* SNPs influence prosociality and how it interacts with gender differences, this research may promote our understanding of the genetic and biological roots of prosociality and morality.

## Ethics Statement

This study was carried out in accordance with the recommendations of “Ethical Issues and Body Protection Guidelines in Psychology, Committee for Protecting Human and Animal Subjects, School of Psychological and Cognitive Sciences at Peking University”; with written informed consent from all subjects. All subjects gave written informed consent in accordance with the Declaration of Helsinki. The protocol was approved by the “Committee for Protecting Human and Animal Subjects, School of Psychological and Cognitive Sciences at Peking University.”

## Author Contributions

SS, YS contributed to the conception and design of the work. SS collected and analyzed the data. SS, NW, YS contributed to the writing of the manuscript.

## Conflict of Interest Statement

The authors declare that the research was conducted in the absence of any commercial or financial relationships that could be construed as a potential conflict of interest.
