# Supplementary material for: How Oxytocin Receptor (OXTR) Single Nucleotide Polymorphisms Act on Prosociality: The Mediation Role of Moral Evaluation
Source: Front Psychol. 2017 Mar 21;8:396. doi: 10.3389/fpsyg.2017.00396 (PMC5359230; doi:10.3389/fpsyg.2017.00396)
Supplement: Supplementary file 1 [file Data_Sheet_1.DOCX]

In the present study, moral evaluation of social behaviors had been assessed with two kinds of behavior, namely moral (prosocial) and immoral (antisocial) ones. We do split the data and get similar findings.

The effects of rs2254298, gender, and their interactions on evaluation of each kind of behaviors are tested separately. Results of ANOVA analyses are shown in Table S2. There was a signiﬁcant interaction between gender and rs2254298 on evaluation of prosocial behavior, *F*(2, 784) = 6.19, *p* = .002, partial *η^2^* = 0.016. Simple eﬀect tests showed that only in males did carriers of these three genotypes diﬀer in moral evaluation of prosocial behavior [*Bonferroni* test: *F* (2, 784) = 5.71, *p* = .003, partial *η^2^* = 0.014]. Speciﬁcally, G allele carriers (GA and GG) evaluated prosocial behaviors more affirmatively (GA vs. AA: *p* = 0.003, Cohen’s *d* = 0.620; GG vs. AA: *p* = 0.030, Cohen’s *d* = 0.433)

And there was a significant interaction between gender and rs2254298 on moral evaluation of antisocial behavior, *F*(2, 784) = 4.08, *p* = .0176, partial *η^2^* = 0.010. Simple eﬀect tests showed that only in males did these three genotype carriers diﬀer (though marginally) in moral evaluation of antisocial behavior [*Bonferroni* test: *F* (2, 784) = 2.51, *p* = .082, partial *η^2^* = 0.006]. Speciﬁcally, comparing to AA carriers, GA carriers evaluated immoral behaviors more negatively, though marginally (*p* = 0.079, Cohen’s *d* = 0.399).

Moral evaluation of prosocial (*r* = 0.301, *p* < 0.001) and antisocial behavior (*r* = 0.259, *p* < 0.001) are both positively related to prosocial tendency. Multiple regression was then used to investigate whether signiﬁcant influence from gender, rs2254298 and their interaction on moral evaluation were carried onto the endorsement on prosocial behaviors. Prosocial tendency and two kinds of moral evaluation were predicted by gender, rs2254298 genotype, and their interaction in a pattern consistent with the ANOVA results (see Tables S3 and Tables S4). Furthermore, interaction of rs2254298 G allele and gender on prosocial tendency was reduced when moral evaluation of each kind of behavior was included in the model, 95% *CI* = [0.042, 0.276] for moral evaluation of prosocial behavior and 95% *CI* = [0.014, 0.193] for moral evaluation of antisocial behavior.

However, when we test the mediation effect by gender, some differences were found. Moral evaluation of prosocial behavior mediated the role of rs2254298 on prosocial tendency in both genders, 95% *CI* = [0.016, 0.225] for males and [-0.077, -0.004] for females. But moral evaluation of antisocial behavior could not act as mediator, 95% *CI* = [-0.015, 0.148] for males and [-0.069, 0.000] for females).

**Table S1. Prosocial tendency and moral evaluation by genotype and gender.**

|  |  | females | |  | males | |  | total | |
| --- | --- | --- | --- | --- | --- | --- | --- | --- | --- |
| Moral  Evaluation |  | *n* | *M(SD)* |  | *n* | *M(SD)* |  | *n* | *M(SD)* |
| Prosocial  Behaviors | AA | 43 | 5.22 (0.66) |  | 22 | 4.24 (1.27) |  | 65 | 4.89 (1.02) |
|  | GA | 211 | 5.03 (0.76) |  | 119 | 4.91 (0.85) |  | 330 | 4.99 (0.80) |
|  | GG | 280 | 4.95 (0.81) |  | 115 | 4.75 (1.08) |  | 395 | 4.89 (0.90) |
| Antisocial  Behaviors | AA | 43 | 5.03 (0.85) |  | 22 | 3.92 (1.44) |  | 65 | 4.65 (1.19) |
|  | GA | 211 | 4.73 (0.91) |  | 119 | 4.43 (1.09) |  | 330 | 4.63 (0.99) |
|  | GG | 280 | 4.78 (0.90) |  | 115 | 4.32 (1.20) |  | 395 | 4.65 (1.01) |

**Table S2. ANOVA results for the interaction of genotype and gender on prosocial tendency and moral evaluation.**

|  | **Genotype × Gender** | | |  | **Genotype** | | |  | **Gender** | | |
| --- | --- | --- | --- | --- | --- | --- | --- | --- | --- | --- | --- |
| Moral Evaluation | *F* | *p* | *η^2^_p_* |  | *F* | *p* | *η^2^_p_* |  | *F* | *p* | *η^2^_p_* |
| Prosocial Behaviors | 6.19 | 0.002 | 0.016 |  | 2.63 | 0.073 | 0.007 |  | 24.80 | <0.001 | 0.031 |
| Antisocial Behaviors | 4.08 | 0.017 | 0.010 |  | 0.31 | 0.733 | 0.001 |  | 37.19 | <0.001 | 0.045 |

**Table S3. Regression analysis with gender, rs2254298A, rs2254298G, and moral evaluation of prosocial behavior predicting prosocial tendency**

|  |  | **Moral evaluation of**  **prosocial behavior** | | | **Prosocial Tendency** | | |
| --- | --- | --- | --- | --- | --- | --- | --- |
|  |  | *β* | *t* | *ΔR^2^* | *β* | *t* | *ΔR^2^* |
| step 1 | |  |  | **0.015** |  |  | 0.001 |
|  | gender | -0.12 | -3.52*** |  | 0.03 | 0.84 |  |
| step 2 | |  |  | 0.004 |  |  | <0.001 |
|  | rs2254298A | 0.07 | 1.75 |  | 0.01 | 0.32 |  |
|  | rs2254298G | 0.03 | 0.90 |  | 0.02 | 0.56 |  |
| step 3 | |  |  | **0.015** |  |  | **0.017** |
|  | rs2254298A×gender | 0.02 | 0.59 |  | 0.03 | 0.83 |  |
|  | rs2254298G×gender | 0.13 | 3.48** |  | 0.14 | 3.65*** |  |
| step 4 | |  |  | - |  |  | **0.086** |
|  | Moral evaluation of  prosocial behavior | - | - |  | 0.30 | 8.68*** |  |

Note. **p* < 0.05, ***p* < 0.01, ****p* < 0.001.

**Table S4. Regression analysis with gender, rs2254298A, rs2254298G, and moral evaluation of antisocial behavior predicting prosocial tendency**

|  |  | **Moral evaluation of**  **antisocial behavior** | | | **Prosocial Tendency** | | |
| --- | --- | --- | --- | --- | --- | --- | --- |
|  |  | *β* | *t* | *ΔR^2^* | *β* | *t* | *ΔR^2^* |
| step 1 | |  |  | **0.042** |  |  | 0.001 |
|  | gender | -0.20 | -5.84*** |  | 0.03 | 0.84 |  |
| step 2 | |  |  | <0.001 |  |  | <0.001 |
|  | rs2254298A | 0.01 | 0.15 |  | 0.01 | 0.32 |  |
|  | rs2254298G | -0.01 | -0.14 |  | 0.02 | 0.56 |  |
| step 3 | |  |  | **0.010** |  |  | **0.017** |
|  | rs2254298A×gender | 0.04 | 1.05 |  | 0.03 | 0.83 |  |
|  | rs2254298G×gender | 0.10 | 2.85** |  | 0.14 | 3.65*** |  |
| step 4 | |  |  | - |  |  | **0.068** |
|  | Moral evaluation of  antisocial behavior | - | - |  | 0.27 | 7.61*** |  |

Note. **p* < 0.05, ***p* < 0.01, ****p* < 0.001
